# *FTO *variant rs9939609 is associated with body mass index and waist circumference, but not with energy intake or physical activity in European- and African-American youth

**DOI:** 10.1186/1471-2350-11-57

**Published:** 2010-04-09

**Authors:** Gaifen Liu, Haidong Zhu, Vasiliki Lagou, Bernard Gutin, Inger S Stallmann-Jorgensen, Frank A Treiber, Yanbin Dong, Harold Snieder

**Affiliations:** 1 Unit of Genetic Epidemiology and Bioinformatics, Department of Epidemiology, University Medical Center Groningen, University of Groningen, Groningen, The Netherlands; 2 Georgia Prevention Institute, Department of Pediatrics, Medical College of Georgia, Augusta, GA, USA

## Abstract

**Background:**

Genome-wide association studies found common variants in the fat mass and obesity-associated (*FTO*) gene associated with adiposity in Caucasians and Asians but the association was not confirmed in African populations. Association of *FTO *variants with insulin resistance and energy intake showed inconsistent results in previous studies. This study aimed to assess the influence of *FTO *variant rs9939609 on adiposity, insulin resistance, energy intake and physical activity in European - (EA) and African-American (AA) youth.

**Methods:**

We conducted a cross-sectional study in EA and AA youths. One thousand, nine hundred and seventy-eight youths (48.2% EAs, 47.1% male, mean age 16.5 years) had measures of anthropometry. Percent body fat (%BF) was measured by dual-energy X-ray absorptiometry, visceral adipose tissue (VAT) and subcutaneous abdominal adipose tissue (SAAT) by magnetic resonance imaging. Energy intake and physical activity were based on self report from up to 7 24-hour recalls. Physical activity was also measured by accelerometry.

**Results:**

*FTO *rs9939609 was significantly associated with body mass index (BMI) (*P *= 0.01), weight (*P *= 0.03) and waist circumference (*P *= 0.04), with per-allele effects of 0.4 kg/m^2^, 1.3 kg and 0.8 cm, respectively. No significant association was found between rs9939609 and %BF, VAT, SAAT or insulin resistance (*P *> 0.05), or between rs9939609 and energy intake or vigorous physical activity (*P *> 0.05). No significant interactions of rs9939609 with ethnicity, gender, energy intake or physical activity were observed (*P *> 0.05).

**Conclusions:**

The *FTO *variant rs9939609 is modestly associated with BMI and waist circumference, but not with energy intake or physical activity. Moreover, these effects were similar for EAs and AAs. Improved understanding of the effect of the *FTO *variant will offer new insights into the etiology of excess adiposity.

## Background

Obesity is becoming an increasingly important clinical and public health challenge worldwide and is associated with several comorbidities such as type 2 diabetes, cardiovascular diseases, metabolic syndrome and certain forms of cancer [1-3]. Obesity results from the combined effects of genes, environment and life style[4]. In this context, an understanding of the effects of life style and genes on obesity and also their interactions is important to provide a basis for determining the role they could have on the development and prevention of obesity.

Lifestyle factors, including diet and physical inactivity, are important contributors to weight gain and obesity. However, previous studies showed inconsistent results regarding the association of obesity with physical activity[5,6] or energy intake[7,8].

Genetic factors play an important role in the development of obesity. The identification of susceptibility genes for obesity, especially common genetic variants in the general population, therefore, is of great importance to improve prediction and preventive efforts. Two independent genome-wide association (GWA) studies firstly discovered significant associations of common genetic variants (rs9939609) in the fat mass and obesity-associated (*FTO*) gene [9,10] with body mass index (BMI) as a measure of general obesity. Simultaneously a third study identified the effect of *FTO *on obesity while testing for population stratification [11]. The associations of *FTO *and obesity-related phenotypes were further replicated in various populations including Caucasians and Asians [12-14], but could not be confirmed in an African population[15]. The *FTO *gene was also reported to be associated with fasting glucose and insulin, but additional adjustment for BMI abolished the association in some studies[16,17], but not in others[18,19].

The function of *FTO *remains incompletely understood. Because this gene is expressed particularly in the brain, skeletal muscle and adipose tissue, *FTO *may be associated with fatness through effects on regulation of energy homeostasis in the hypothalamus[20]. Two recent studies suggested that the polyporphisms of the *FTO *gene were associated with energy intake rather than energy expenditure[21,22]. However, other studies suggested there is no association of *FTO *with either energy intake or energy expenditure (e.g., physical activity)[23,24].

The main objectives of this study were, first, to assess whether the previously identified common variant rs9939609 in the *FTO *gene is associated with adiposity and insulin resistance in African-American (AA) and European-American (EA) youth available from the Georgia Cardiovascular Twin study[25], the Lifestyle, Adiposity and Cardiovascular Health in Youths (LACHY) study[8] and the Adiposity Prevention through Exercise (APEX) study[26]; second, to investigate the potential interaction of rs9939609 with ethnicity, gender or lifestyle behaviors(diet and physical activity); third, to investigate whether rs9939609 has a direct influence on energy intake and physical activity. In addition to anthropometric measures, more accurate indices for adiposity such as visceral adipose tissue (VAT) and subcutaneous abdominal adipose tissue (SAAT) measured by magnetic resonance imaging (MRI), and percentage of body fat (%BF) based on dual-energy X-ray absorptiometry (DXA) were used in our study.

## Methods

### Subjects

The present study included 1978 subjects from 3 cohorts, the Georgia Cardiovascular Twin study [(n = 1210 twins with 588 monozygotic (MZ) (291 pairs and 6 singletons) and 622 dizygotic (DZ) twins (285 pairs and 52 singletons)], the LACHY study (n = 525, including 38 sib-pairs) and the APEX study (n = 243, including 29 sib-pairs). All twins in the Georgia Cardiovascular Twin study[25]were recruited from public middle and high schools in the Augusta, Georgia area and the cohort consisted of roughly equal numbers of AAs and EAs (56.1% EA, 47.2% male, mean age [standard deviation (SD)]: 18.1 [3.8] years). All twin pairs were reared together and zygosity was determined by genotyping 5 standard microsatellite markers using buccal swabs or buffy coat DNA[27]. The LACHY study consisted of approximately equal numbers of EA and AA boys and girls (52.8% EA, 43.8% male) aged 14-18 years recruited from high schools in the Augusta, Georgia area[8]. In the APEX study, subjects were AA boys and girls only (53.6% male), aged 8 to 12 years recruited from local elementary schools. Subjects eligible for the study were only those that weighted <136.1 kg[26]. The criteria for classifying subjects as AAs or EAs using self-identification of ethnicity have been described previously[28]. Subjects in all the 3 studies were apparently healthy, free of any acute or chronic illness on the basis of parental reports and were taking no medication that could influence the results. The Institutional Review Board at the Medical College of Georgia approved the studies. Informed consent was obtained from all subjects and by parents if subjects were <18 years of age.

### Anthropometrics and body composition assessment

Height and weight were measured by standard methods using a wall-mounted stadiometer and a digital scale, respectively. BMI was calculated as weight/height^2 ^(kg/m^2^). Waist circumference (in cm) was measured twice at the center of the umbilicus over the T-shirt and the values were averaged. Skinfold thicknesses (i.e. triceps, subscapular, and suprailiac) were measured on the right side of the body with Lange calipers according to established protocols[29]. Three sets of measurements for each skinfold were recorded and averaged. The inter-correlations were >99%. Measurements of skinfold thickness were available in 1976 subjects. BMI and the sum of the 3 skinfold thicknesses were used as measures of general adiposity, while waist circumference was used as a measure of central adiposity.

### Biochemical assays

Fasting glucose and insulin concentrations were measured at the NIDDK supported Clinical Nutrition Research Unit Core Laboratory at the University of Alabama. Glucose was measured in 10 μL of sera using an Ektachem DT II system (Johnson and Johnson Clinical Diagnostic, Rochester, NY). Insulin was assayed in duplicate 100-μL aliquots of serum by specific radioimmunoassay (Linco Research, Inc, St Charles, Mo). Cross-reactivity with proinsulin is <0.2%. Assay sensitivity was 3.41 mU/mL. The intra-assay coefficient of variation was 3.7%. Fasting glucose and insulin were only available in a subsample of twins as twins coming on afternoon visits were not required to fast.

Based on fasting glucose and insulin we used the homeostasis model assessment (HOMA) 2 to calculate insulin resistance (HOMA2-IR) and beta-cell function (HOMA2-%B) using a nonlinear computer model as specified in the HOMA2 software http://dtu.ox.ac.uk/homa.

### Dual-energy X-ray absorptiometry

In the LACHY study, %BF was measured using DXA (Hologic QDR-4500W, software version 6.0, Waltham, MA, USA). DXA provides reliable values for %BF[8]. Repeat measurements were performed using the QDR-4500W machine with 219 adolescents and the intraclass correlation coefficient (ICC) for %BF was found to be 0.99. For some subjects, DXA values were only available from the Hologic QDR-1000W, but not from the Hologic QDR-4500W model. For these individuals, %BF values were derived from prediction equations based on 284 youths who were assessed on both instruments, using linear regression; ethnicity, gender and the QDR-1000W measurement were the predictor variables. The multiple R^2 ^value for %BF was 0.99[30]. In the APEX study, all %BF measurements were obtained using a Hologic QDR-1000 (Waltham, MA) as previously described, the ICC for %BF was > 0.998 between two scans[31]. DXA calibration was done each day, as specified by the Hologic Company. DXA scans were not performed in the Georgia Cardiovascular Twin study.

### Magnetic resonance imaging

In both the LACHY and APEX studies, VAT and SAAT was determined using MRI (1.5 T General Electric Medical Systems, Milwaukee, WI) as described previously[32]. Briefly, with subjects in the supine position, a series of five, 1-cm-thick, transverse images was acquired beginning at the inferior border of the fifth lumbar vertebra and proceeding toward the head. A gap is left between the slices to avoid cross-talk. Values for VAT and SAAT from a single image were calculated in terms of surface area (cm^2^) and the volume (cm^3^) estimated by multiplying the surface area by the image width (1 cm) and then summing the five images. VAT and SAAT were measured in the Department of Radiology on equipment dedicated to patient care. VAT and SAAT measures were obtained in those subjects who underwent testing on days when the MRI equipment was available for the research study. Eventually, VAT and SAAT measurements were available for 394 subjects. Measurements of VAT and SAAT were not available in the Georgia Cardiovascular Twin study and in the males of the APEX study.

### Environmental variables

Free-living diet was measured with individual, non-consecutive, 24-h recalls that covered the period from midnight to midnight of the previous day. In the LACHY study, we sought to obtain seven recalls from each participant, one of each day of the week and only those subjects that provided at least four recalls were included in the analysis. The diet assessment has been previously described in detail[8]. In the APEX study, two 24-h diet recalls were obtained from each participant. Physical activity was self-reported and quantified using our modified version of the previous day physical activity recall[26], which recorded activities in 30-min time blocks for 24-h period (midnight to midnight). Subjects were asked to recall the activities concurrently with each 24-h diet recall and report at which level of effort (light, moderate, hard, very hard) they engaged in each activity. In the LACHY study, physical activity was measured with MTI Actigraph monitors (model 7164; MTI Health Services, Fort Walton Beach, FL)[33]. The daily mean number of hours spent in vigorous physical activity (VPA) rather than those spent in moderate physical activity was included in the present study, since only VPA was previously shown to be negatively associated with %BF[8]. No measurements of free-living diet or physical activity were available in the Georgia Cardiovascular Twin study.

### Genotyping

DNA was extracted from buffy coats by using the QiaAmp DNA Blood Mini Kit (Qiagen, Valencia, CA) or from buccal swabs by using QuickExtract DNA Extraction Kit (Epicentre, Madison, WI). The *FTO *rs9939609 was genotyped by allelic discrimination Taqman assays (Applied Biosystems, Foster City, CA). PCR were performed in a 96-well format in a total of 5 μl reaction volume using 10 ng of genomic DNA and FAM/VIC dye labeled allelic probes with the Taqman Universal Fast Master mix and subjected to 95°C for 15 min, and 40 cycles of 95°C for 15 sec and 60°C for 1 min on an ABI 9800 Fast Thermocycler (Applied Biosystems, Foster City, CA). The Taqman assay plates were transferred to an ABI 7500 Fast Real Time PCR system in which the fluorescence intensity in each well of the plate was recorded and genotypes were analyzed using Sequence Detection Software 1.3. Genotyping quality control procedures included genotyping 10% duplicates for accuracy checking and inclusion of both positive and non-template controls in each 96-well plate. Genotyping success rate was 99.5% for rs9939609. Genotyping accuracy as determined by concordance between duplicates was 100%.

### Statistical Analyses

The main effects of the SNP on obesity-related phenotypes were tested using structural equation modeling with the statistical software Mx[34]. In this approach a model is specified for both the means and the variance-covariance matrices. We adapted the model described previously[35] to include MZ twin pairs, DZ twin pairs(or sib-pairs) and unpaired twins/singletons, which allows for non-independent observations in twin and family data. By modeling MZ and DZ (or sib-pair) covariances separately we accounted for their different degrees of relatedness. The SNP effect was analyzed in the combined data from all 3 cohorts. Effects of age, ethnicity, gender and cohort were regressed out for all variables before using the residuals in Mx models. Gender and ethnicity-specific effects of the SNP were modeled as interactions of the SNP with gender and/or ethnicity using regression analyses within a generalized estimating equations (GEE) framework, which takes the non-independency of twin and family data into account and yields unbiased *P *values[36]. Furthermore, the associations between rs9939609 and energy intake, physical activity, as well as the interaction of rs9939609 with energy intake or physical activity on adiposity-traits in the LACHY and APEX studies including sib-pairs only were tested using GEE.

In the APEX study, subjects were randomized to a physical activity intervention or control group. Obesity-related phenotypes were measured three times, at the baseline, mid- and end-point of the study. The measurement at the baseline, prior to randomization, was used for the analysis.

Hardy-Weinberg equilibrium (HWE) and ethnic differences in allele and genotype frequencies were tested by a *χ*^2 ^test in only one member per family (i.e, twins or sib pairs), which was chosen randomly to prevent inflated significance. All variables except %BF were log transformed to obtain better approximations of the normal distribution. Preliminary and GEE analyses were performed using Stata 10 software (StataCorp, College Station, TX). A *P *value of ≤ 0.05 was considered to be statistically significant.

## Results

### Participant characteristics

Descriptive statistics for age, height, adiposity and insulin resistance related variables and environmental predictors of adiposity in combined data of the 3 studies are presented by ethnicity and gender in Table 1. Effects of ethnicity, gender and their interaction were tested using GEE with age and cohort identifier included as covariates. The mean age of the total sample was 16.5 years. Many significant gender differences were observed, although some of these were limited to one ethnic group. Similarly, many of the significant ethnic differences were limited to either males or females, as indicated in Table 1.

**Table 1:** General characteristics of study subjects

	European-American (EA)				African-American (AA)				Ethnicity *P* (a/b)	Gender *P* (a/b)
			
	male		female		male		female			
			
	N	mean (SD)	N	mean (SD)	N	mean (SD)	N	mean (SD)		
Age, years	475	17.4(3.4)	478	17.8(3.4)	457	15.2(4.4)	568	15.8(4.5)	0.52	0.19
**Anthropometry and adiposity**										
Height (cm)	475	173.4(9.0)	478	162.6(6.9)	457	165.2(16.0)	568	158.4(10.8)	0.67	< 0.001
Weight(kg)	475	69.9(18.2)	478	60.9(15.6)	457	63.8(22.3)	568	62.6(21.1)	< 0.001**	< 0.001
BMI(kg/m^2^)	475	23.1(5.1)	477	22.8(4.9)	457	22.8(5.4)	566	24.5(6.6)	< 0.001**	< 0.001^##^
Waist circumference (cm)	474	80.4(12.9)	476	75.2(12.8)	457	74.6(13.5)	568	75.9(14.9)	0.02*	< 0.001^#^
Suprailiac skinfold (mm)	474	14.7(9.2)	478	18.7(9.7)	457	14.6(11.4)	568	20.7(12.0)	0.001**	< 0.001
Subscapular skinfold (mm)	474	13.2(7.8)	478	16.7(8.5)	457	14.7(9.5)	567	20.3(11.1)	< 0.001**	< 0.001
Triceps skinfold (mm)	475	13.1(7.1)	477	20.5(7.4)	457	13.6(8.3)	568	21.6(9.7)	0.74	< 0.001
Sum of skinfolds (mm)	475	41.0(22.8)	477	55.9(24.0)	457	42.9(28.1)	567	62.7(31.3)	0.001**	< 0.001
%BF	132	18.7(7.8)	144	29.4(7.1)	227	20.9(10.0)	265	29.5(8.5)	0.34	< 0.001
VAT(cm^3^)	64	95.8(56.2)	72	111.9(55.6)	70	67.0(49.6)	188	100.8(72.6)	< 0.001*	< 0.001
SAAT (cm^3^)	64	622.9(556.1)	72	903.4(517.2)	70	613.1(671.8)	187	1027.3(807.7)	0.05**	< 0.001
**Insulin resistance**										
Fasting glucose (mmol/L)	164	5.3(0.5)	175	5.0(0.5)	192	5.3(0.6)	284	5.0(0.5)	0.35/0.86	< 0.001/< 0.001
Fasting insulin (pmol/L)	163	100.8(57.2)	169	94.2(58.0)	186	102.6(55.6)	282	131.9(76.4)	< 0.001**/< 0.001**	< 0.001^##^/< 0.001^##^
HOMA2-%B	160	130.3(48.4)	169	137.4(50.0)	186	134.3(51.2)	281	174.3(64.2)	< 0.001**/< 0.001**	< 0.001^##^/< 0.001^##^
HOMA2-IR	160	1.9(1.0)	169	1.7(1.0)	186	1.9(1.0)	281	2.4(1.3)	< 0.001**/< 0.001**	< 0.001^##^/< 0.001^##^
**Environmental variables**										
EI(Kilojoules/d)	134	9627.9 (2373.8)	146	7292.2 (2214.1)	233	8109.3 (2809.8)	268	6939.0 (2356.7)	0.006*/0.005*	< 0.001/< 0.001
%CARBO	134	53.4(6.3)	146	55.8(6.2)	233	51.0(7.2)	269	53.5(6.9)	0.001/0.004	< 0.001/< 0.001
%PRO	134	14.4(2.8)	146	13.6(2.7)	233	14.4(3.0)	269	13.3(2.8)	0.39/0.15	< 0.001/< 0.001
%FAT	134	32.8(4.8)	146	32.0(5.2)	234	35.2(5.8)	268	34.1(5.1)	< 0.001/< 0.001	0.009/0.008
VPA (hours/d)	134	0.8(0.7)	146	0.4(0.5)	213	0.7(0.7)	257	0.3(0.5)	0.28/0.29	< 0.001/< 0.001

BMI = body mass index; SD = standard deviation; %BF = percentage of body fat; VAT = visceral adipose tissue; SAAT = subcutaneous abdominal adipose tissue; HOMA2-%B = homeostasis model assessment 2 beta-cell function; HOMA2-IR = homeostasis model assessment 2 insulin resistance; EI = energy intake; %CARBO= percentage of energy from carbohydrates; %PRO= percentage of energy from protein; %FAT= percentage of energy from fat; VPA = vigorous physical activity.

N: number of subjects with phenotype and genotype data;

a: adjusted for age and cohort identifier; b: adjusted for age, cohort identifier and BMI

*significant only in males, **significant only in females, ^#^significant only in EAs, ^##^significant only in AAs.

### Environmental predictors of adiposity

Table 2 shows the regression analysis in LACHY and APEX studies combined (n = 784) for BMI, waist circumference, sum of skinfolds, %BF, VAT and SAAT. After adjustment for age, ethnicity, gender, cohort and the interaction between ethnicity and gender, VPA was negatively related to sum of skinfolds (*P *= 0.01), %BF (*P *< 0.001) and SAAT (*P *= 0.05) accounting for 0.43%, 2.35% and 1.11% of the variance respectively, but not to BMI (*P *= 0.96), waist circumference (*P *= 0.83) or VAT (*P *= 0.45). Energy intake was a negative determinant of BMI (*P *= 0.03), sum of skinfolds (*P *= 0.02), %BF (*P *< 0.001), VAT (*P *= 0.02) and SAAT (*P *= 0.01) accounting for 0.61%, 0.74%, 1.82%, 1.16% and 1.21% of the variance respectively. Percentage of energy from carbohydrates (%CARBO) was negatively associated with BMI (*P *= 0.02), sum of skinfolds (*P *= 0.008) and %BF (*P *= 0.007) (Table 2). Percentage of energy from protein (%PRO) was a positive predictor of BMI (*P *< 0.001), waist circumference (*P *< 0.001), sum of skinfolds (*P *< 0.001) and %BF (*P *< 0.001). The relationship of dietary percentage of energy from fat (%FAT) with these dependent variables was not significant (Table 2).

**Table 2:** Environmental predictors of BMI, waist circumference, sum of skinfolds, %BF, VAT and SAAT in the combined LACHY and APEX studies

	BMI			Waist circumference			Sum of skinfolds			%BF			VAT			SAAT		
	
	*β*	*P*	%V	*β*	*P*	%V	*β*	*P*	%V	β	P	%V	*β*	*P*	%V	*β*	*P*	%V
VPA (h/day)	-	0.96	-	**+**	0.83		**-**	**0.01**	0.43	-	** < 0.001**	2.35	-	0.45	-	-	**0.05**	1.11
EI (Kilojoules/d)	**-**	**0.03**	0.61	**-**	0.84		**-**	**0.02**	0.74	-	** < 0.001**	1.82	**-**	**0.02**	1.16	-	**0.01**	1.21
%CARBO	-	**0.02**	0.48	-	0.09		-	**0.008**	0.78	-	**0.007**	0.73	-	0.96	-	-	0.21	-
%PRO	**+**	** < 0.001**	1.84	+	** < 0.001**	1.19	+	** < 0.001**	1.47	+	** < 0.001**	1.29	**+**	0.81	-	+	0.09	-
%FAT	+	0.44	-	+	0.66		+	0.20	-	+	0.18	-	-	0.84	-	+	0.40	-

Based on the model with age, gender, ethnicity, cohort and the interaction of gender and ethnicity. Negative and positive indicate the direction of relation between the predictor and dependent variables. *P *< 0.05 are indicated in bold. %V = explained variance.

BMI = body mass index; %BF = percentage of body fat; VAT = visceral adipose tissue; SAAT = subcutaneous abdominal adipose tissue; VPA = vigorous physical activity; EI = energy intake; %FAT = percentage of energy from fat; %CARBO = percentage of energy from carbohydrates; %PRO= percentage of energy from protein.

### Allele and genotype frequencies

Rs9939609 was common in both ethnic groups with minor allele frequency (MAF) of 44.6% in EA and 48.4% in AA. There was a significant difference in allele frequencies between EA and AA subjects (*P *= 0.03), but no significant difference in genotype frequencies (*P *= 0.08). Rs9939609 was in HWE in both ethnic groups (*P *= 0.34 in EA and 0.92 in AA).

### Associations between *FTO *rs9939609 and adiposity-relatedphenotypes

**Figure 1:**
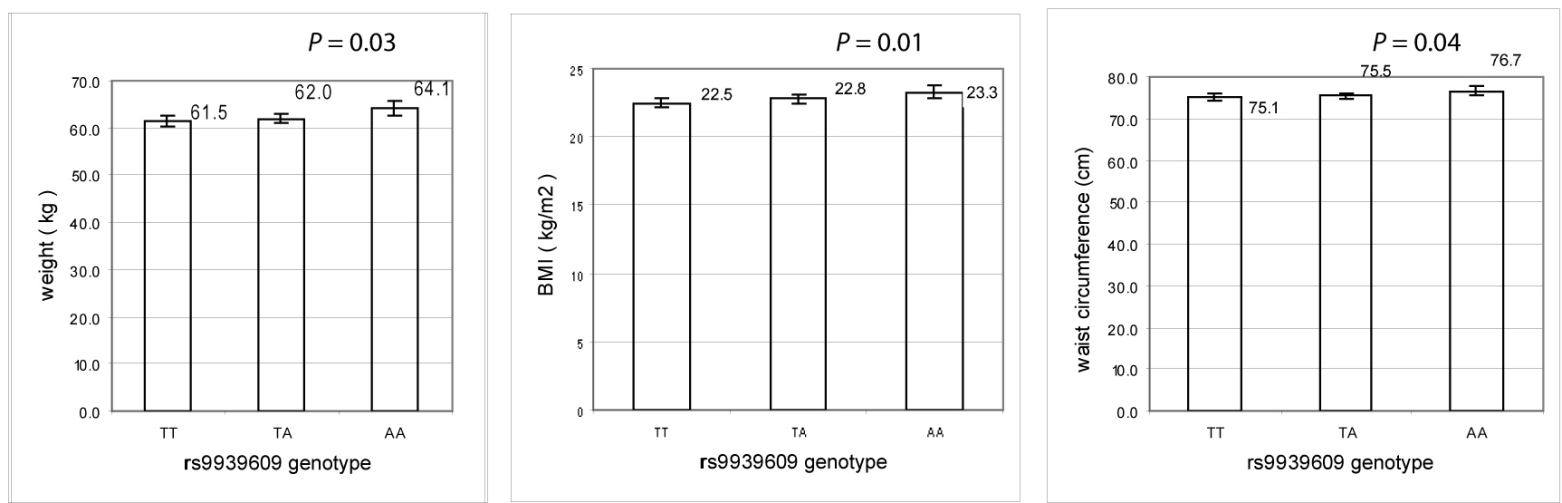
**Association between rs9939609 and weight, BMI and waist circumference**. (1) Weight, BMI and waist circumference were presented as geometric mean and 95%CI adjusted for age, ethnicity, gender and cohort; (2) *P *values represent significance of the additive model, adjusted for age, gender, ethnicity and cohort identifier.

We found modest association between rs9939609 and BMI (per-allele effect of 0.4 kg/m^2^, *P *= 0.01), weight (per-allele effect of 1.3 kg, *P *= 0.03), and waist circumference (per-allele effect of 0.8 cm, *P *= 0.04) in additive models (Figure 1). The explained percentages of variance were 0.24%, 0.20% and 0.16%, respectively. In a recessive model, rs9939609 was also significantly associated with BMI (*P *= 0.03), weight (*P *= 0.006), and waist circumference (*P *= 0.02), with explained percentages of variance of 0.17%, 0.22% and 0.17%, respectively. The A allele carriers showed higher sum of skinfold thicknesses, %BF, VAT and SAAT compared to the TT allele carriers, but the difference did not reach statistical significance (Table 3). No significant associations were found between rs9939609 and fasting glucose, fasting insulin, HOMA2-%B or HOMA2-IR before or after additional adjustment for BMI (Table 3). No significant interactions between rs9939609 and ethnicity or gender were observed for any of these adiposity-related phenotypes (*P *> 0.05). However, we further tested the associations between rs9939609 and adiposity-related phenotypes in EAs and AAs separately. We found that in both EAs and AAs, the A allele carriers showed higher weight, BMI and waist circumference compared to the TT allele carriers, although the associations were significant (*P *= 0.02, 0.02 and 0.01, respectively) only in AAs (additional file 1: Table S1).

**Table 3:** Association between rs9939609 and adiposity-related phenotypes

Variables	No.	Mean (95%CI)			χ^2^ (a/b)	P (a/b)	Variance (%)
			
	TT/AT/AA	TT	TA	AA			
Weight(kg)	599/962/417	61.5(60.4-62.6)	62.0(61.0-63.0)	64.1(62.6-65.7)	4.97	**0.03**	**0.20**
BMI(kg/m^2^)	599/962/414	22.5(22.2-22.9)	22.8(22.5-23.1)	23.3(22.8-23.8)	6.38	**0.01**	**0.24**
Waist circumference (cm)	599/960/416	75.1(74.3-76.0)	75.5(74.8-76.2)	76.7(75.6-77.9)	4.39	**0.04**	**0.16**
Suprailiac (mm)	599/962/417	14.4(13.7-15.0)	14.4(13.9-15.0)	14.7(13.7-15.6)	1.07	0.30	-
Subscapular(mm)	598/961/417	14.1(13.5-14.7)	14.1(13.6-14.6)	14.7(13.9-15.5)	2.28	0.13	-
Triceps(mm)	599/961/417	15.7(15.2-16.3)	15.6(15.1-16.1)	15.8(15.0-16.6)	0.37	0.55	-
Sum of skinfold(mm)	599/960/417	44.8(43.2-46.5)	44.9(43.4-46.4)	46.0(43.7-48.5)	1.24	0.27	-
%BF	183/377/208	24.9(23.7-26.1)	25.1(24.2-25.9)	25.2(24.0-26.4)	0.09	0.77	-
VAT(cm^3^)	97/187/110	78.0(69.5-87.5)	82.1(75.2-89.8)	78.3(68.9-89.0)	0.00	0.98	-
SAAT (cm^3^)	97/187/109	627.1(539.1-729.6)	661.6(587.2-745.4)	673.5(568.4-798.0)	0.69	0.41	-
Fasting glucose(mmol/L)	213/391/211	5.07(5.02-5.13)	5.10(5.06-5.15)	5.10(5.05-5.16)	0.57/0.25	0.45/0.62	-
Fasting insulin (pmol/L)	208/387/205	94.4(88.6-100.6)	99.7(94.8-104.9)	94.8(86.5-103.9)	0.66/0.19	0.42/0.67	-
HOMA2-%B	206/385/205	140.1(134.8-145.7)	142.4(138.1-146.8)	139.3(132.5-146.4)	0.01/1.09	0.92/0.30	-
HOMA2-IR	206/385/205	1.7(1.6-1.9)	1.8(1.8-1.9)	1.8(1.6-1.9)	0.62/0.18	0.43/0.67	-

BMI = body mass index; %BF = percentage of body fat; VAT = visceral adipose tissue; SAAT = subcutaneous abdominal adipose tissue; HOMA2-%B = homeostasis model assessment 2 β-cell function; HOMA2-IR = homeostasis model assessment 2 insulin resistance; P-values represent significance of the additive model (per-allele effect); Significant associations (*P *≤ 0.05) are indicated in bold.

All variables are presented as means and 95%CI adjusted for age, gender, ethnicity and cohort identifier.

%variance = 100* (the difference of R-square value of the regression model additional with SNP compared to the base model).

a: adjusted for age, ethnicity, gender, cohort identifier;

b: adjusted for age, ethnicity, gender, cohort identifier and BMI.

### Associations of *FTO *rs9939609 with energy intake and VPA in LACHY and APEX studies combined

No significant association was found between rs9939609 and the total energy intake, %CARBO, %PRO or %FAT. Neither did we find association of rs9939609 with VPA (irrespective of adjustment for BMI), whether assessed through self-report in the combined LACHY and APEX studies or accelerometry in the LACHY study (Table 4).

No significant gene-environment interactions between rs9939609 and energy intake or VPA were observed for any of the adiposity or insulin resistance-related phenotypes (*P *> 0.05) (data not shown).

**Table 4:** Association of rs9939609 with energy intake, percentage of energy intake from carbohydrates, protein and fat and vigorous physical activity measured through recall and accelerometry

Variables	No.	Mean (SD)			P (a/b)
		
	TT/AT/AA	TT	TA	AA	
EI(Kilojoules/d)	187/379/209	7958.8(2772.2)	7706.8(2698.3)	7883.9(2470.2)	0.75/0.79
%CARBO	187/379/210	53.3(7.3)	53.2(6.9)	53.2(6.7)	0.83/0.90
%PRO	187/380/209	13.9(2.9)	13.7(2.9)	14.2(2.8)	0.34/0.41
%FAT	186/380/210	33.6(5.3)	34.1(5.6)	33.5(5.2)	0.91/0.89
VPA (hours/d)^c^	181/359/204	0.5(0.6)	0.5(0.6)	0.6(0.6)	0.63/0.62
VPA (mins/d)^d^	123/221/129	4.8(7.5)	5.3(7.2)	3.9(7.2)	0.26

*P*-values represent significance of the additive model (per-allele effect);

EI = energy intake; %CARBO = percentage of energy from carbohydrates; %PRO = percentage of energy from protein; %FAT= percentage of energy from fat; VPA = vigorous physical activity.

a: adjusted for age, ethnicity, gender;

b: adjusted for age, ethnicity, gender and BMI;

c: recalled VPA in the LACHY and APEX study;

d: measured VPA through accelerometer in the LACHY study.

## Discussion

In the current study, the association between a common variant in the *FTO *gene and adiposity and insulin resistance-related phenotypes was investigated in 1978 EA and AA youths, available from the Georgia Cardiovascular Twin, LACHY and APEX studies. We replicated the association between *FTO *variant rs9939609 and BMI, weight and waist circumference, which were modestly significant. Effects of *FTO *rs9939609 did not depend on ethnicity or gender. No significant associations of rs9939609 with fasting glucose, fasting insulin or insulin resistance were found, irrespective of correction for BMI; Associations of rs9939609 with energy intake or vigorous physical activity was not significant, nor were effects of rs9939609 on adiposity modified by energy intake or vigorous physical activity.

In the LACHY and APEX studies, VPA was a negative predictor of sum of skinfolds, %BF and SAAT, and energy intake was a strong negative determinant of all dependent variables except waist circumference. The inverse relation between energy intake and adiposity could be explained by the fact that individuals performing more free-living physical activity tend to have less fat accumulation but at the same time higher energy intake. The dietary %PRO was a positive predictor for BMI, waist circumference, sum of skinfolds and %BF, but the dietary %FAT was not found to be a predictor of any obesity-related phenotype. This finding is in line with a previous study on 6-14 year old children, which showed that higher %PRO was associated with overweight after adjusting for age, gender and total energy, whereas %FAT was not found to be a predictor of BMI[37]. We found an inverse association between %CARBO and BMI, sum of skinfolds and %BF, which is consistent with a previous finding[38]. However, it remains difficult to resolve cause and effect in cross-sectional studies, in which energy intake and adiposity are measured at the same time.

Since Frayling et al[10] first reported the significant associations between the *FTO *variant rs9939609 and adiposity-related phenotypes such as BMI, weight, waist circumference, %BF and skinfolds in both children and adults, several studies have replicated these findings in Europeans[13,16] and Asians[12]. In our sample of EA and AA youth, we replicated the significant associations of rs9939609 with BMI, weight and waist circumference using an additive model, although a recessive model was more appropriate for weight and waist circumference. Our per-A allele effect of 0.4 kg/m^2 ^in BMI is similar to the effect that Frayling reported in UK children at the age of 11 years (0.4 kg/m^2^, *P *= 7 × 10^-9^) and a little higher than that in Finnish children at the age of 14 years (0.1 kg/m^2^, *P *= 0.04). We found that the variance in BMI explained by rs9939609 was 0.24%, which is lower than previously reported ~1.0%[10]. In addition, overall per-A allele increases in weight (~1.3 kg) and waist circumference (~0.8 cm) observed in our study were similar to the effects reported by Frayling et al[10](~1.2 kg and ~1.0 cm, respectively). Similar to Frayling et al[10], we found that sum of skinfolds increased with the number of A allele carried; however, the difference did not reach significance. The lack of association between rs9939609 and %BF is not in agreement with Frayling et al's finding in children at 9 years of age[10]. The discrepancy in the findings between the two studies could be partly due to differences in population characteristics, such as age, gender or ethnic composition, as well as environmental exposures. Although the A-allele carriers had slightly higher VAT and SAAT compared to TT carriers, we did not find significant associations between rs9939609 and VAT or SAAT. This might due to the smaller number of subjects that had MRI examinations.

Previous studies reported associations of rs9939609 with fasting glucose, fasting insulin, or insulin resistance. In most of these studies adjustment for BMI abolished the associations[16,17], but in some studies they remained significant[18,19]. We did not find any significant associations before or after adjustment for BMI.

The *FTO *gene is significantly associated with weight, BMI and other adiposity-related phenotypes. The mechanism underlying the association of *FTO *gene and adiposity may be due to a functional effect of *FTO *itself [39]. It is known that *FTO *is highly expressed in the hypothalamic region[40], an area that is known to be involved in the regulation of appetite; and studies on the expression of *FTO *suggest it might have a role in central control of energy homeostasis[20,39]. Previous studies that have investigated the association of rs9939609 with energy intake and energy expenditure, physical activity, all showed lack of association of rs9939609 with energy expenditure or physical activity [21-23], but findings on the associations with energy intake were inconsistent[21,22,24,41,42]. We also investigated the role of rs9939609 in the control of energy intake and physical activity in the LACHY and APEX studies, in which measures on VPA and energy intake were available. No significant association was found between rs9939609 and self-reported VPA. Similarly, no significant association was found between rs9909609 and VPA measured through accelerometry. Furthermore, we estimated energy expenditure through physical activity, weight and metabolic equivalents assigned to different activity categories. No significant association was found between rs9939609 and energy expenditure (data not shown), which is in line with previous studies that did assess energy expenditure directly through more sophisticated means[23,41]. We did not find significant association between rs9939609 and energy intake, which is in line with the findings of Hakanen et al [24] and Sonestedt et al [42], but inconsistent with the findings of other studies [21,22,41,43]. A study in 2726 Scottish children found that rs9939609 does not appear to be involved in the regulation of energy expenditure, but may have a role in the control of food intake and food choice[41]. In 3337 UK children, Wardle et al found that the rs9939609 A allele is likely to exert its effects by influencing appetite[44] and the T allele might be protective against overeating by promoting responsiveness to internal signals of satiety[43]. Currently little is known about the function of the *FTO *gene. Functional tests in mice indicated that *FTO *may play a role in adipocyte function but not adipogenesis[45]. Wåhlén et al [46]studied *FTO *with regard to fat cell function and adipose tissue gene expression, their results suggested that *FTO *might be involved in body weight regulation through lipolysis. In our study, we did not observe significant association between rs9939609 and energy intake. However, based on our current sample size, the power to detect 1% of the total variance for energy intake is only about 70%. Thus, the non-significant associations should be interpreted with caution, and will provide useful information for future meta-analysis efforts.

Previous studies reported that low physical activity accentuated the effect of rs9939609 on body fat accumulation[47]. However, we did not find any interaction of rs9939609 with physical activity on any phenotype related to adiposity, which is in line with a number of other studies[48,49]. In our study, contrary to Sonestedt et al's finding [42], we did not observe a significant interaction of rs9939609 with fat or carbohydrate intake on BMI, which might be due to our smaller sample size.

Studying populations of different ancestry will help to globally identify and understand the genetic and environmental factors associated with obesity. As such, we included AAs as well as EAs in our study. The MAF in EAs (0.45) in our study was the same as that reported in HapMap, while the MAF in AAs (0.48) was slightly lower (0.52 in HapMap). The MAF in EAs was a little higher than reported in another study[10]. The association between rs9939609 and BMI has been firmly established in Europeans[9-11,14], but the findings in populations of African ancestry are inconsistent [15,50]. In 604 middle-aged AAs, rs9939609 was significantly associated with BMI and waist circumference (*P *= 0.014 and 0.034, respectively)[50], which is in line with our findings. However, this association could not be confirmed in a native African population[15], This might represent a true lack of association, but it is also possible that any effect of *FTO *genotype on adiposity is of limited relevance in a lean population where little excess food is available compared to a similar ethnic population in an obesogenic environment. In a large sample of African Americans, no significant association was found between adiposity and *FTO *variant rs8050136, which is in high LD with rs9939609 (r^2 ^= 0.82 in YRI population)[13]. We found significant association between rs9939609 and BMI, weight and waist circumference in the combined EA and AA samples. Although the MAF in EAs was significantly different from those in AAs, no interaction between rs9939609 and ethnicity was observed for weight, BMI or other adiposity-related phenotypes. Thus, our findings suggest that the effects of rs9939609 on obesity-related phenotypes were similar for EAs and AAs. For example, the per-allele effect on BMI was 0.35 kg/m^2 ^and 0.45 kg/m^2 ^in EAs and AAs, respectively. This finding provides useful information in understanding the role of *FTO *variants in adiposity of multi-ethnic populations.

The major strengths of our study are (1) the inclusion of in-depth estimates based on multiple self reports of both dietary energy intake and physical activity, which allowed us to investigate the direct association with rs9939609 and their potential interactions with the SNP in their effect on adiposity; and (2) the use of %BF by DXA and VAT and SAAT by MRI, which are more precise measures of general and/or central adiposity than BMI and waist circumference. Furthermore, the inclusion of AA as well as EA youth allowed us to investigate a potential interaction of rs9939609 with ethnicity. Several limitations of our study need to be acknowledged as well. In the Georgia Cardiovascular Twin study, no measurements of %BF, VAT and SAAT were available since DXA scans and MRI examinations were not performed. Meanwhile, in the twin study, fasting glucose and insulin were only available from part of the subjects since fasting was not requested for twins examined in the afternoon. Sexual maturation was not assessed in the twin cohort and could not be incorporated as a covariate. A final limitation is that our recall measures of free-living diet and physical activity were based on self report and not measured directly.

## Conclusion

In summary, we replicated in a large sample of EA and AA youth the significant association between the *FTO *variant rs9939609 and BMI, weight and waist circumference, and these effects are similar for EAs and AAs. Moreover, we did not find any significant association between rs9939609 and energy intake or physical activity. Furthermore, we did not observe any significant interactions between rs9939609 and gender, energy intake or physical activity for any adiposity-related phenotypes. These findings may be helpful in improving our understanding of the underlying mechanism and pathways whereby the variant influences the development of adiposity.

## Abbreviations

GWAS: genome-wide association study; SNPs: single nucleotide polymorphisms; FTO: fat mass and obesity-associated; AA: African-American; EA: Europe-American; LACHY: the Lifestyle, Adiposity and Cardiovascular Health in Youths; APEX: Adiposity Prevention through Exercise; BMI: Body Mass Index; %BF: percent body fat; DXA: dual-energy X-ray absorptiometry; VAT: visceral adipose tissue; SAAT: subcutaneous abdominal adipose tissue; MRI: magnetic resonance imaging; MZ: monozygotic; DZ: dizygotic; HOMA: homeostasis model assessment; ICC: intraclass correlation coefficient; IR: insulin resistance; HOMA2-%B: homeostasis model assessment 2 β-cell function; GEE: generalized estimating equations; HWE: Hardy-Weinberg equilibrium; MAF: minor allele frequencies; CI: confidence interval; VPA: vigorous physical activity; EI: energy intake; %CARBO: percentage of energy from carbohydrates; %PRO: percentage of energy from protein; %FAT: percentage of energy from fat.

## Competing interests

The authors have indicated they have no financial relationship to this article to disclose, and there is no conflict of interest associated with this work.

## Authors' contributions

GFL performed the statistical analysis and drafted the manuscript; HDZ did the genotyping and participated in the drafting of the manuscript. VL participated in the drafting of the manuscript. BG conducted the LACHY study and edited the manuscript. ISS edited the manuscript. FAT conducted the twin study and edited the manuscript. YBD provided significant advice and participated in the drafting of the manuscript. HS developed the original idea for the study and participated in the design of the study and in the drafting of the manuscript. All authors read and approved the final manuscript.

## Pre-publication history

The pre-publication history for this paper can be accessed here:

http://www.biomedcentral.com/1471-2350/11/57/prepub

## Supplementary Material

Additional file 1 Supplementary table S1. Click here for file
