# Supplementary material for: FTO variant rs9939609 is associated with body mass index and waist circumference, but not with energy intake or physical activity in European- and African-American youth
Source: BMC Med Genet. 2010 Apr 9;11:57. doi: 10.1186/1471-2350-11-57 (PMC2864242; doi:10.1186/1471-2350-11-57)
Supplement: Additional file 1 — Supplementary table S1. [file 1471-2350-11-57-S1.DOC]

Additional file 1

Table S1 Association between rs9939609 and obesity-related phenotypes in EAs and AAs

| Variables | No. | Mean (95%CI) | | | χ^2^  (a/b) | *P*  (a/b) | Variance  (%) |
| --- | --- | --- | --- | --- | --- | --- | --- |
|  | TT/AT/AA | TT | TA | AA |  |  |  |
| Weight(kg) |  |  |  |  |  |  |  |
| EA | 322/442/189 | 63.3(61.8-64.9) | 63.1(61.8-64.5) | 65.2(63.2-67.4) | 0.08 | 0.78 | **-** |
| AA | 277/520/228 | 59.8(58.2-61.4) | 60.8(59.4-62.2) | 63.2(61.1-65.4) | 5.55 | **0.02** | **0.32** |
| BMI(kg/m^2^) |  |  |  |  |  |  |  |
| EA | 322/442/188 | 22.3(21.8-227) | 22.5(22.1-22.9) | 23.0(22.3-23.6) | 1.02 | 0.31 | **-** |
| AA | 277/520/226 | 22.7(22.2-23.3) | 23.1(22.7-23.6) | 23.6(22.9-24.3) | 5.35 | **0.02** | **0.28** |
| Waist circumference (cm) |  |  |  |  |  |  |  |
| EA | 322/440/188 | 76.9(75.7-78.1) | 76.6(75.6-77.6) | 78.0(76.3-79.7) | 0.00 | 0.97 | **-** |
| AA | 277/520/228 | 73.5(72.3-74.8) | 74.4(73.3-75.4) | 75.8(74.2-77.5) | 6.82 | **0.01** | **0.38** |
| Suprailiac (mm) |  |  |  |  |  |  |  |
| EA | 322/442/189 | 14.4(13.6-15.2) | 14.4(13.7-15.2) | 14.5(13.4-15.8) | 0.23 | 0.63 | **-** |
| AA | 277/520/228 | 14.2(13.1-15.3) | 14.5(13.6-15.5) | 15.0(13.6-16.5) | 0.78 | 0.38 | **-** |
| Subscapular(mm) |  |  |  |  |  |  |  |
| EA | 321/442/189 | 13.2(12.6-13.9) | 13.1(12.5-13.7) | 13.6(12.6-14.6) | 0.21 | 0.64 | **-** |
| AA | 277/519/228 | 14.7(13.8-15.7) | 15.2(14.4-16.0) | 16.0(14.8-17.2) | 2.24 | 0.13 | **-** |
| Triceps(mm) |  |  |  |  |  |  |  |
| EA | 322/441/189 | 15.3(14.7-16.0) | 15.3(14.7-15.9) | 16.0(15.1-17.0) | 0.83 | 0.36 | **-** |
| AA | 277/520/227 | 16.0(15.2-16.9) | 16.0(15.3-16.7) | 16.0(14.9-17.2) | 0.02 | 0.89 | **-** |
| Sum of skinfolds | |  |  |  |  |  |  |
| EA | 322/441/189 | 43.6(41.6-45.6) | 43.4(41.7-45.3) | 44.9(42.1-48.0) | 0.40 | 0.53 | **-** |
| AA | 277/519/227 | 45.7(43.1-48.5) | 46.5(44.3-48.8) | 47.6(44.2-51.4) | 0.80 | 0.37 | **-** |
| %BF |  |  |  |  |  |  |  |
| EA | 64/124/88 | 26.1(24.1-28.0) | 23.4(22.5-25.1) | 23.6(22.1-25.1) | 4.17 | 0.04 | 0.90 |
| AA | 119/253/120 | 24.4(22.9-25.9) | 24.3(23.3-25.4) | 24.1(22.3-26.0) | 2.14 | 0.14 | **-** |
| VAT(cm^3^) |  |  |  |  |  |  |  |
| EA | 32/58/46 | 103.6(86.4-124.3) | 90.1(79.2-102.6) | 87.1(73.6-103.1) | 1.65 | 0.20 | **-** |
| AA | 65/129/64 | 64.1(54.6-75.2) | 76.5(67.2-86.9) | 72.5(60.1-87.6) | 0.97 | 0.32 | **-** |
| SAAT (cm^3^) |  |  |  |  |  |  |  |
| EA | 32/58/45 | 686.3(561.4-839.1) | 617.6(521.7-731.0) | 640.3(531.0-772.2) | 0.10 | 0.76 | **-** |
| AA | 63/126/61 | 595.8(477.8-742.8) | 693.3(585.2-821.3) | 770.9(623.8-952.6) | 1.90 | 0.17 | **-** |
| Fasting glucose(mmol/L) |  |  |  |  |  |  |  |
| EA | 87/156/96 | 5.1(5.1-5.2) | 5.1(5.1-5.2) | 5.2(5.1-5.3) | 0.00/0.00 | 0.99/0.98 | **-** |
| AA | 126/235/115 | 5.0(5.0-5.1) | 5.1(5.0-5.1) | 5.1(5.0-5.1) | 0.69/0.15 | 0.41/0.70 | **-** |
| Fasting insulin  (pmol/L) |  |  |  |  |  |  |  |
| EA | 85/152/95 | 84.2(73.7-96.4) | 90.0(83.5-97.0) | 82.6(75.2-90.8) | 0.60/0.47 | 0.44/0.49 | **-** |
| AA | 123/235/110 | 100.6(93.0-108.8) | 106.6(99.8-113.8) | 109.8(98.6-122.6) | 3.50/1.66 | 0.06/0.20 | **-** |
| HOMA2-%B |  |  |  |  |  |  |  |
| EA | 84/150/95 | 128.7(120.6-137.4) | 130.4(124.1-137.0) | 123.7(116.7-131.0) | 0.92/0.87 | 0.34/0.35 | **-** |
| AA | 122/235/110 | 148.0(140.8-155.5) | 150.7(145.0-156.6) | 153.1(142.1-164.8) | 2.15/0.63 | 0.14/0.43 | **-** |
| HOMA2-IR |  |  |  |  |  |  |  |
| EA | 84/150/95 | 1.6(1.4-1.8) | 1.7(1.6-1.8) | 1.5(1.4-1.7) | 0.80/0.60 | 0.37/1.81 | **-** |
| AA | 122/235/110 | 1.8(1.7-2.0) | 2.0(1.8-2.1) | 2.0(1.8-2.2 | 3.55/0.44 | 0.06/0.18 | **-** |

EA=European-American; AA=African-American; BMI = body mass index; %BF=percentage of body fat; VAT=visceral adipose tissue; SAAT=vubcutaneous abdominal adipose tissue; HOMA2-%B=homeostasis model assessment 2 β-cell function; HOMA2-IR=homeostasis model assessment 2 insulin resistance; *P*-values represent significance of the additive model (per-allele effect); significant associations (*P*≤0.05) are indicated in bold.

All variables are presented as means and 95%CI adjusted for age, gender and cohort identifier.

%variance=100* (the difference of R-square value of the regression model additional with SNP compared to the base model).

a: adjusted for age, gender, cohort identifier; b: adjusted for age, gender, cohort identifier and BMI.
